# Nuclear transport of paxillin depends on focal adhesion dynamics and FAT domains

**DOI:** 10.1242/jcs.172643

**Published:** 2016-05-15

**Authors:** Aneesh R. Sathe, G. V. Shivashankar, Michael P. Sheetz

**Affiliations:** 1 Mechanobiology Institute, National University of Singapore, Singapore 117411; 2 Department of Biological Sciences, National University of Singapore, Singapore 117543; 3 IFOM-NUS Joint Research Laboratory; 4 Department of Biological Sciences, Columbia University, New York, NY 10027, USA

**Keywords:** FAT domain, Focal adhesion, Nucleus, Paxillin

## Abstract

The nuclear transport of paxillin appears to be crucial for paxillin function but the mechanism of transport remains unclear. Here, we show that the nuclear transport of paxillin is regulated by focal adhesion turnover and the presence of FAT domains. Focal adhesion turnover was controlled using triangular or circular fibronectin islands. Circular islands caused higher focal adhesion turnover and increased the nuclear transport of paxillin relative to triangular islands. Mutating several residues of paxillin had no effect on its nuclear transport, suggesting that the process is controlled by multiple domains. Knocking out FAK (also known as PTK2) and vinculin caused an increase in nuclear paxillin. This could be reversed by rescue with wild-type FAK but not by FAK with a mutated FAT domain, which inhibits paxillin binding. Expressing just the FAT domain of FAK not only brought down nuclear levels of paxillin but also caused a large immobile fraction of paxillin to be present at focal adhesions, as demonstrated by fluorescence recovery after photobleaching (FRAP) studies. Taken together, focal adhesion turnover and FAT domains regulate the nuclear localization of paxillin, suggesting a possible role for transcriptional control, through paxillin, by focal adhesions.

## Abstract (precis)

**Summary:** We find that nuclear translocation of paxillin is tuned by focal adhesion maturation. This could provide a method for mechanosensing signals to be used by cells to control transcription.

## INTRODUCTION

Members of the LIM domain family of proteins, such as zyxin, Trip6, Hic-5 and paxillin, play a role in the mechanosensitive control of transcription (Kadrmas and Beckerle, 2004; Mammoto et al., 2012; Wang and Gilmore, 2003). Mechanisms that facilitate the nuclear transport of a number of these proteins have been identified, including the stretch dependency of zyxin (Cattaruzza et al., 2004; Lele et al., 2006) and the redox sensitivity of Hic-5 (Shibanuma et al., 2005). However, signals that control nuclear transport of paxillin have not yet been described.

Paxillin is a LIM domain scaffold protein that localizes at focal adhesions and also cycles through the nucleus (Brown and Turner, 2004). In prostate cancer cells, paxillin controls the transcription of cyclin D1 through c-Fos (Sen et al., 2012, 2010). Cell proliferation is probably promoted by paxillin, most likely by inhibiting the expression of the H19 transcript (Dong et al., 2009). As a focal adhesion scaffold protein, paxillin has a large interactome. Thus, the nuclear cycling of paxillin could transfer mechanical information sensed at focal adhesions to the nucleus, where it influences transcription.

Nuclear export of paxillin is blocked by leptomycin-B (LMB), an inhibitor of CRM1 (also known as exportin 1)-based nuclear export (Kudo et al., 1999). How the protein cycles between the nucleus and cytoplasm is, however, less clear. Paxillin associates with the RNA-binding protein poly(A)-binding protein-1 (PABP1, also known as PABPC1), but only to facilitate PABP1 nuclear export (Woods et al., 2005, 2002). Furthermore, although phosphorylation of the paxillin LD4 motif is a signal for nuclear export, it is not required for import (Dong et al., 2009). Paxillin lacks a canonical nuclear import sequence (NIS) and for this reason it has been proposed that there is a carrier complex for its nuclear transport (Brown and Turner, 2004; Deakin and Turner, 2008). Suggested partners include c-Abl, PABP-1 and FAK (also known as PTK2) (Dong et al., 2009; Lewis and Schwartz, 1998); however, in each case, a role in the nuclear transport of paxillin has not been fully explored.

Here, we discuss an undiscovered pathway that controls the nuclear transport of paxillin. We show that paxillin transport to the nucleus depends on the cellular geometry, which affects focal adhesion dynamics. We also show that nuclear transport of paxillin is increased by knocking out FAK or vinculin, whereas the overexpression of focal-adhesion-targeting (FAT) domains reduces nuclear transport. These results indicate a role for focal adhesion stability in the nuclear transport of paxillin.

## RESULTS AND DISCUSSION

### Nuclear accumulation of paxillin depends on cell geometry but not on matrix composition

**Fig. 1.**
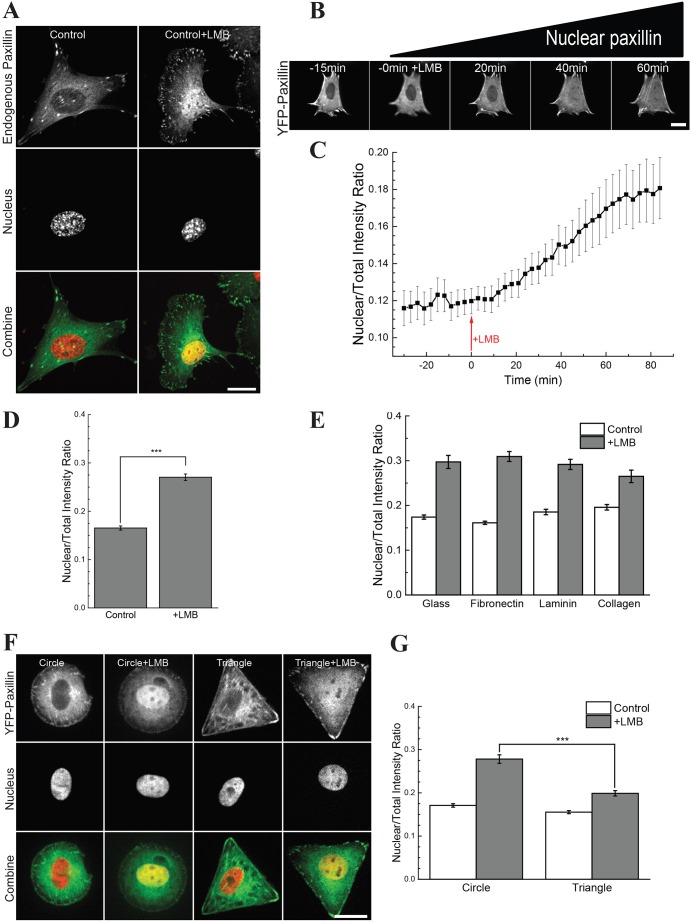
**Nuclear accumulation of paxillin depends on cell geometry but not on matrix composition.** (A,D) Images and quantification (NTIR) of endogenous paxillin with and without LMB (*n*>50). (B,C) Images and NTIR of a time-series of cells expressing YFP–paxillin, with LMB added at 0 min (15 min after start of imaging) (*n*>10). (E) NTIR for cells grown on uncoated glass, and glass coated with fibronectin, laminin and collagen (*n*>30). (F,G) Cells grown on circular or triangular fibronectin islands. Paxillin is shown in green, the nucleus is shown in red and yellow indicates colocalization. Quantitative results are mean±s.e.m. ***P<0.001 (Welch's *t*-test). Scale bars: 20 µm.

A standard image-analysis-based assay was developed to measure the nuclear accumulation of paxillin. To enrich the nuclear signal, we used LMB (Dong et al., 2009; Woods et al., 2002). In order to compare nuclear paxillin in cells with varying expression levels, we used the nuclear to total intensity ratio (NTIR) as a standard measure. Normally, ∼10% of paxillin was found in the nucleus. After 1 h of LMB treatment, we observed that ∼27% of endogenous paxillin accumulated in the nucleus (Fig. 1A,D). Similar results were seen with Crm inhibitor III (Fig. S1A). Western blotting experiments supported the image analysis results (Fig. S1C,D). The image analysis was further validated by using zyxin (Nix et al., 2001) as a reporter of nuclear translocation (Fig. S1E,F). In live observations, YFP–paxillin was detectable in the nucleus after ∼20 min of LMB treatment, and a substantial rise was observed after 45 min (Fig. 1B). The half-time for nuclear accumulation was 30 min (Fig. 1C). The lower NTIR of live samples compared to fixed cells was likely caused by the photo-sensitivity of LMB.

To broadly probe the molecular processes behind the nuclear transport of paxillin, we tested the effect of different matrix proteins. Fibronectin, laminin and collagen coatings were tested, as well as an uncoated glass control, but no significant difference in the NTIR was observed (Fig. 1E). For further experiments, fibronectin-coated substrates were used. Overall, these observations showed that LMB treatment produced a paxillin NTIR of ∼0.25, with a half-time of 30 min, and this was independent of matrix composition.

The nuclear translocation of paxillin could also be altered by modulating focal adhesion dynamics because paxillin is a prominent focal adhesion scaffolding protein. Treating cells with Y27632 (Fig. S3A–C), to destabilize focal adhesions, or using active RhoA (RhoV14) (Fig. S3E,F) caused small changes in the NTIR. This suggests that healthy focal adhesion dynamics are important for the nuclear transport of paxillin. To investigate this, we altered focal adhesion dynamics by modifying the cell geometry using fibronectin-coated micropatterns (Chen et al., 1998). Triangular micropatterns with an area of 1600 µm^2^ were compared to circular micropatterns of the same size. These were used with the intent of stimulating the formation of stronger focal adhesions at the corners of the triangular micropatterns (Sero et al., 2011; Théry and Bornens, 2006; Théry et al., 2006a,b). We observed that the focal adhesion turnover rate on circular patterns was twice that of triangular patterns (Fig. S1G,H). Using cells transfected with YFP–paxillin, we probed the nuclear transport of paxillin and found that cells on the circular patterns had an NTIR of 0.27, whereas cells on the triangular patterns had an NTIR of 0.17 (Fig. 1F,G). Overall this indicates that the nuclear transport of paxillin depends on the dynamics of focal adhesions.

### Site-specific phosphorylation does not affect the nuclear transport of paxillin

**Fig. 2.**
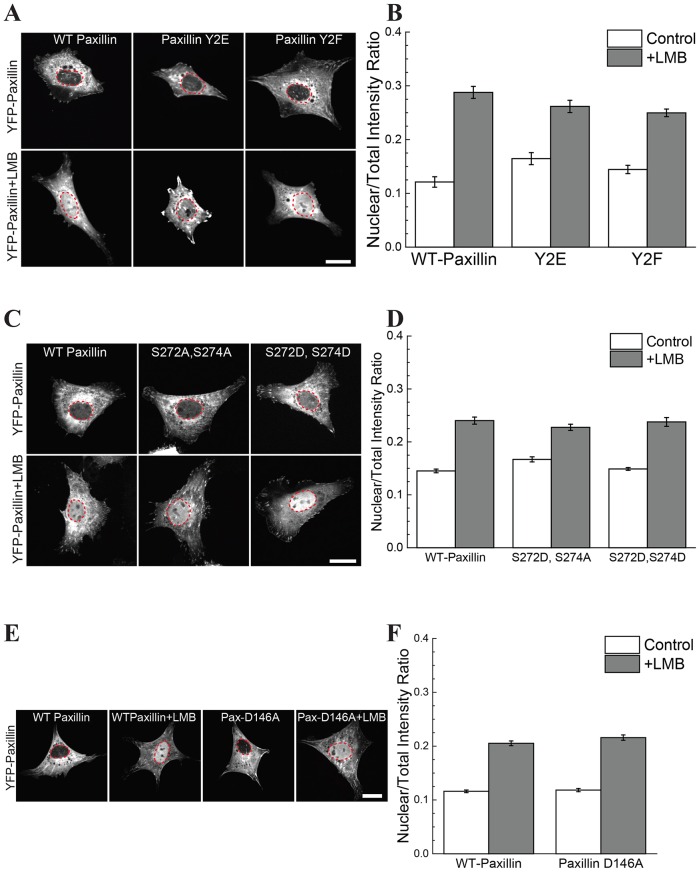
**Site-specific phosphorylation does not control nuclear transport of paxillin.** (A,B) Images and NTIR of YFP–paxillin mutated at Y31 and Y118. Y2E represents the phospho-mimetic double mutant Y31E and Y118E; Y2F represents the non-phosphorylatable double mutant, Y31F and Y118F. No significant change in nuclear transport was observed between WT and either of the paxillin mutants. (C,D) YFP–paxillin with the S272A,S274A or S272D,S274D double mutations was transfected and no change in nuclear transport was observed. (E,F) Images and NTIR of YFP–paxillin with a D146A mutation; no change in nuclear transport was observed. Paxillin is gray; the nucleus is outlined in red. Quantitative results are mean±s.e.m.; *n*>50 for each condition. Scale bars: 20 µm.

Given that focal adhesion turnover rates affected the nuclear transport of paxillin, we investigated whether the phosphorylation sites Y31 and Y118, which are known to affect focal adhesion turnover (Zaidel-Bar et al., 2007), would also cause a change in the nuclear transport of paxillin. Compared to the non-phosphorylatable (Y2F) and the phospho-mimetic (Y2E) paxillin constructs, no significant change in the nuclear transport of paxillin was observed (Fig. 2A,B).

It has been suggested that the LD2 and LD4 motifs of paxillin are important for its nuclear cycling (Dong et al., 2009). The S272 and S274 residues in the LD4 motif modify paxillin–GIT1 association (Schmalzigaug et al., 2007), and phosphorylation of S272 might block nuclear export (Dong et al., 2009). We generated S272A, S274A and S272D, S274D double paxillin point mutants to test whether these sites had an effect on nuclear transport; however, no differences were observed (Fig. 2C,D). A mutation of paxillin in the LD2 motif (D146A) is known to affect paxillin–FAK binding (Scheswohl et al., 2008). However, this mutation also had no effect on the nuclear cycling of paxillin. Taken together, these results indicate that the LD2 and LD4 domains do not act alone in their control of nuclear transport.

### FAK and vinculin regulate nuclear transport of paxillin through the FAT domain

Focal adhesion stabilization depends on the phosphorylation of paxillin by FAK and the recruitment of vinculin (Pasapera et al., 2010; Thomas, 1999). Moreover, FAK translocates to the nucleus and controls cell proliferation (Lim et al., 2008). The report that the nuclear presence of paxillin affects the H19 gene, which is also involved in cell proliferation, lends weight to the idea that FAK might be a carrier for paxillin to the nucleus (Dong et al., 2009). FAK binds to paxillin through its C-terminal FAT domain (Hayashi et al., 2002; Richardson and Parsons, 1996; Tachibana et al., 1995). Moreover the four-helix bundle of the FAT domain is similar to that of vinculin (Arold et al., 2002; Hayashi et al., 2002) suggesting that vinculin might control the transport of paxillin in a similar manner.

**Fig. 3.**
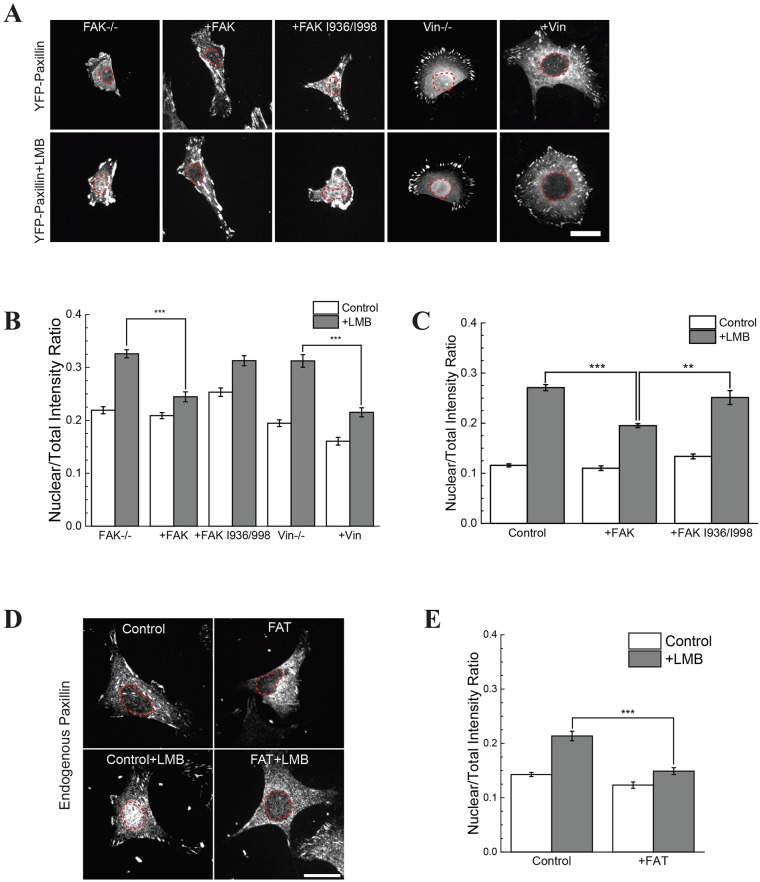
**Nuclear transport of paxillin is controlled by FAK and vinculin through the FAT domain.** (A,B) FAK^−/−^ and vinculin^−/−^ (Vin−/−) cells transfected with YFP–paxillin. FAK^−/−^ cells were rescued with WT-FAK (+FAK) or FAK I936/998 (+FAK I936/998). Vinculin^−/−^ cells were rescued with WT vinculin. (C) Measurement of the NTIR in RPTPα^+/+^ cells transfected with WT-FAK or FAK I936/998. (D,E) RPTPα^+/+^ mouse embryonic fibroblast cells were transfected with GFP–FAT and stained for endogenous paxillin. Quantitative results are mean±s.e.m.; *n*>50 for each condition. **P≤0.01; ***P<0.001 (Welch's *t*-test). Scale bars: 20 µm.

Contrary to expectations, both FAK- and vinculin-knockout cells had increased nuclear paxillin. The FAK^−/−^ and vinculin^−/−^ cells had a NTIR of 0.30 and 0.36, respectively, whereas that of the wild-type (WT) cells was ∼0.2 (Fig. 3A,B). To ensure that the increased nuclear paxillin in FAK^−/−^ cells was not an optical artifact due to the size of the cell, we treated FAK^−/−^ cells with LMB after 30 min of blebbistatin incubation. Although blebbistatin treatment caused substantial cell spreading, there was no change in the nuclear translocation of paxillin in FAK^−/−^ cells (Fig. S2E,F). FAK^−/−^ or vinculin^−/−^ cells rescued with WT FAK or WT vinculin, respectively, reduced the amount of paxillin in the nucleus (Fig. 3A,B). This surprising result indicates that FAK and vinculin actually hamper nuclear transport of paxillin.

Mutating two hydrophobic regions of FAK, I936E and I998E (FAK I936/I998), decreases the interaction between FAK and paxillin (Deramaudt et al., 2014). Disruption of the FAK–paxillin interaction also blocked the restoration of normal nuclear paxillin levels in FAK^−/−^ cells upon FAK expression; that is, transfection with mCherry–FAK-I936/I998 did not decrease the nuclear accumulation of paxillin, whereas expression of WT FAK did (Fig. 3A,B). To determine whether increased levels of FAK in control cells also decreased the nuclear accumulation of paxillin, we expressed WT FAK or FAK I936/998 in control cells with normal FAK levels. WT FAK overexpression decreased levels of nuclear paxillin in control cells, whereas expression of FAK I936/998 did not (Fig. 3C).

Paxillin–FAT domain association could reduce the nuclear transport of paxillin, FAK or vinculin (Alam et al., 2014). To assess this further, we transfected cells with GFP–FAT and performed immunofluorescence staining for paxillin. Non-transfected WT cells showed an NTIR of 0.20 and WT cells transfected with FAT exhibited a reduced nuclear translocation and had an NTIR of 0.15, which is nearly at background levels (Fig. 3D,E). This indicates that the presence of FAT domains is sufficient to inhibit the nuclear transport of paxillin.

### Recovery dynamics of paxillin at focal adhesions are regulated by the FAT domain

**Fig. 4.**
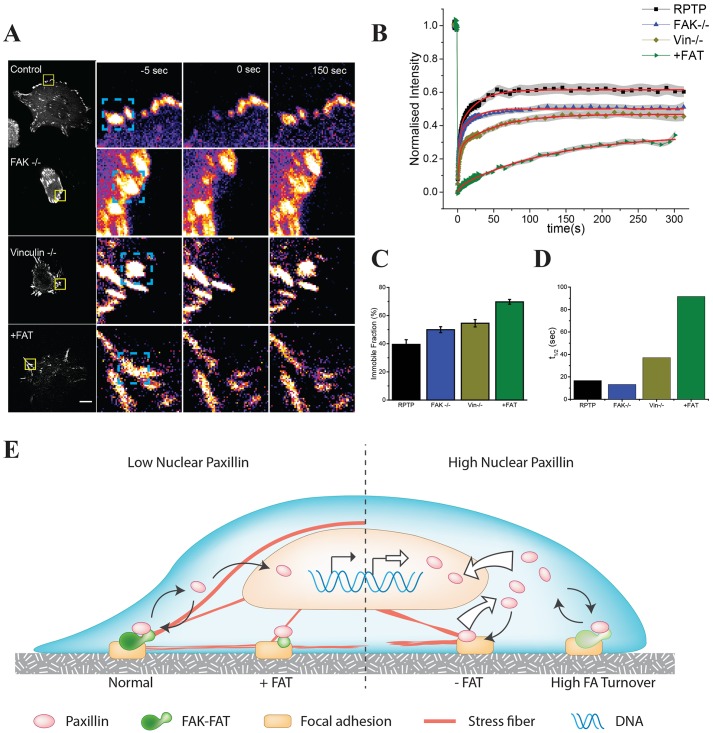
**Paxillin recovery dynamics are controlled by the FAT domain.** (A) Cells transfected with YFP–paxillin were used to study the recovery of paxillin by performing FRAP experiments. The right-hand panels show an example of a bleached focal adhesion (boxed region in left-hand panel) 5 s prior to bleaching, immediately after bleaching and 150 s after bleaching. Scale bar: 10 µm. RPTPα^+/+^ cells were used as the control and for FAT transfection. (B) Quantification of >30 focal adhesions across >10 cells for RPTPα^+/+^, FAK^−/−^, vinculin^−/−^ and RPTPα^+/+^ cells transfected with FAT (+FAT) in >10 cells. The normalized intensity is plotted on the *y*-axis and the gray shadow indicates the s.e.m. of each time point. The red overlay is the curve fitted to the double exponential function. (C,D) The average immobile fraction and half-time of the fitted curve. (E) Model. The left side represents cells with a normal nuclear accumulation of paxillin, normal focal adhesions and excess FAT domains. These cells have normal rates of paxillin exchange at focal adhesion and transport to the nucleus. The right side represents cells with a high nuclear accumulation of paxillin, where cells have a high turnover of focal adhesions or reduced paxillin association with FAT-domain-containing proteins. Increased nuclear paxillin has been linked to transcriptional control of cell proliferation.

FAK and vinculin stabilize focal adhesions and have a higher concentration in stable focal adhesions (Zaidel-Bar et al., 2003). Combined with the information that triangular geometries reduced the amount of nuclear paxillin, we hypothesized that the removal of FAK and vinculin would inhibit focal adhesion, whereas the FAT domain would stabilize focal adhesion and decrease paxillin dynamics. To test this, we performed fluorescence recovery after photobleaching (FRAP) experiments at individual focal adhesions in cells transfected with YFP–paxillin (Fig. 4A).

Paxillin showed a much slower recovery in both FAK^−/−^ and vinculin^−/−^ cells. WT cells had an average recovery half-time of 16 s, whereas in FAK^−/−^ cells the average recovery was 13 s and in vinculin^−/−^ cells it was 37 s (Fig. 4B,D). Rescuing knockout cells caused the recovery dynamics to return towards normal levels (Fig. S4B). Addition of LMB did not significantly affect the recovery dynamics (Fig. S4C). To see whether the overexpression of FAT also had an effect on the behavior of paxillin at focal adhesions, we doubly transfected cells with YFP–paxillin and mCherry–FAT. FRAP of focal adhesions showed that the presence of FAT dramatically reduced the mobile fraction of paxillin, whereas the exchangeable fraction had the same half-time for recovery as the control. The average immobile fraction of YFP–paxillin at focal adhesions was 70% in FAT-expressing cells as compared to 40% in non-transfected cells (Fig. 4B,C). Thus, the FRAP studies combined with the nuclear translocation data indicate that adhesion stability and FAT binding affects the nuclear transport of paxillin.

### Model: association of paxillin with FAT domains at focal adhesions determines its rate of nuclear transport

The data presented above show that FAT domains reduce the mobile fraction of paxillin at focal adhesions and reduce nuclear transport. Conditions such as triangular cell geometry, which provide signals to strengthen adhesions, also reduce the nuclear shuttling of paxillin. LIM domain proteins translocate into the nucleus where they target specific genes. However, no mechanism to control the nuclear transport of paxillin has been proposed. We suggest a model (Fig. 4E), where the FAT-domain-containing proteins aid in the stabilization of focal adhesions and decrease paxillin transport to the nucleus. Given that the binding of paxillin to the adhesion might be through the FAT domain, the interactions of proteins with other domains of FAK and vinculin would enable nuclear transport of paxillin. Consistent with this, overexpression of FAT alone dramatically inhibited nuclear transport by stabilizing adhesions and decreasing the presence of facilitating proteins required for nuclear transport. As adhesion stabilization causes a decrease in paxillin transport to the nucleus, loss of adhesions might enhance the functional roles of paxillin, such as in cancer cell proliferation.

## MATERIALS AND METHODS

### Cell culture and reagents

Dulbecco's modified Eagle's medium (DMEM), RMPI-1640 medium, heat-inactivated fetal bovine serum (HI-FBS), penicillin, streptomycin, HEPES, TrypLE Express (trypsin-like protease), paxillin monoclonal antibody (5H11) and Neon electroporation kits were purchased from Life Technologies (Grand Island, NY, USA). Antibodies used were rabbit anti-LaminB1 (1:5000, cat. no. ab16048, Abcam), goat anti-mouse-IgG (H+L) conjugated to horseradish peroxidase (HRP) (1:5000; cat. no. 1706516, Bio-Rad), goat anti-rabbit-IgG (H+L) conjugated to HRP (1:5000, cat. no. 1720109, Bio-Rad) and mouse anti-α-tubulin, (1:5000, cat. no. T6199, Sigma) antibodies. Hoechst 33342 and LMB were purchased from Sigma. Fibroblast cells were grown in DMEM supplemented with 10% (v/v) HI-FBS, 100 U/ml penicillin and 100 µg/ml streptomycin in 37°C incubators with 5% CO_2_. Nuclear extraction was carried out using the NE-PER nuclear and cytoplasmic extraction kit purchased from ThermoFisher Scientific. Electroporation was used for transient transfection. RPTPα^+/+^ mouse embryonic fibroblasts (Su et al., 1999) were generous gifts from Jan M. Sap, New York University, New York, NY. YFP-paxillin, mCherry-FAK and mCherry-vinculin constructs were a kind gift from Alexander Bershadsky (Mechanobiology Institute, Singapore) and were used for generating the mutants.

### Microcontact printing

Circles or triangles with an area of 1600 µm^2^ were printed on non-culture-treated Petri dishes using microcontact printing, and polydimethylsiloxane (PDMS) (Sylgard 184; Dow Corning) stamps were prepared (Fink et al., 2007; Vedula et al., 2012). After coating with fibronectin (500 µg/ml; Roche), stamps were pressed onto an uncoated plastic-bottom dish (Ibidi, Munich, Germany). The Petri dishes were treated with 0.2% Pluronic F127 (Sigma) for 2 h followed by PBS rinsing.

### Image acquisition

A Perkin Elmer spinning disk confocal microscope (PerkinElmer UltraVIEW VoX, Waltham, MA) with an UplanSApo 60× (1.20 NA) water immersion objective or an UplanSApo 100× (1.40 NA) oil immersion objective (Olympus, Center Valley, PA, USA) and cooled EMCCD camera (C9100.13, Hamamatsu Photonics, Hamamatsu, Japan) was used for image acquisition. A microscope-attached environmental chamber (37°C and 5% CO_2_) was used for long-term imaging. The camera and microscope settings were kept fixed, to compare different cells.

### Image and statistical analysis

NTIR quantification was performed using a custom-written MATLAB code analyzing 3D stacks of images. The code can be obtained at https://github.com/aneeshsathe/Nucleus-Cell-Ratio. Importing of 3D-image stacks from proprietary microscope formats into MATLAB was facilitated by the Bioformats MATLAB plugin (Linkert et al., 2010). Nuclear and cytoplasmic areas in the image were thresholded using appropriate stains. To measure total cell intensity, the nucleus threshold was added to the cytoplasmic threshold before recording the nuclear and total cell intensities. The nuclear to total intensity ratio (NTIR) was used to measure nuclear localization. The output was then screened for correct segmentation and statistical analysis was performed with a pairwise Welch's *t*-test to account for possible unequal variance between samples. FRAP analysis was performed as outlined previously (McNally, 2008). Briefly, bleach correction for quantification was performed using the reference region of interest (ROI) and whole-cell intensity measurements. The corrected FRAP curves were fitted to the following function: 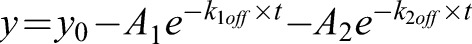, in Origin software. Half-time was calculated as: ln(2)/*k*_*off*_.
